# On the implications of desexualizing vaccines against sexually transmitted diseases: reflections from a practicing pediatrician

**DOI:** 10.1186/s13584-017-0181-0

**Published:** 2017-10-13

**Authors:** Amanda F. Dempsey

**Affiliations:** University of Colorado Denver, 13199 East Montview Blvd, Suite 300, Aurora, CO 80045 USA

**Keywords:** Human papillomavirus, Vaccine, Sexually transmitted infection

## Abstract

Human papillomavirus (HPV) vaccination holds great promise for drastically reducing the incidence of HPV-associated cancers of the genital tract, and possibly also certain head and neck cancers. Unfortunately, rates of HPV vaccine utilization among adolescents are low in many countries. Many research studies have identified the fact that HPV is a sexually transmitted infection as a barrier to higher vaccination rates. This is related to providers’ and parents’ reluctance to discuss or consider the burgeoning sexuality of their child. An approach suggested to overcome this barrier is to “desexualize” the vaccine. This entails focusing discussions and public messages on the cancer-preventing properties of the vaccine and ignoring or minimizing information about HPV’s sexual transmissibility. In an article by Velan and Yadgar, the authors argue that this approach does more harm than good. This associated commentary offers a slightly different viewpoint from one who has been “in the trenches” both clinically and from a research standpoint for many years.

Vaccines against human papillomavirus (HPV) have been available for over a decade and are highly effective at preventing infection with the strains of HPV most likely to cause cancer. While allowing one’s adolescent to receive a vaccine against cancer would seem an obvious choice for many parents, use of the HPV vaccine is far below optimal in many countries. For example, as of 2016 in the US, while 60% of adolescents age 13–17 have started the vaccine series, only 43% have completed it [1]. The question is, why isn’t utilization higher?

Though the reasons parents give for not wanting their adolescent vaccinated are multiple, one concern that has been raised by many is the fact that HPV is a sexually transmitted disease. Some parents and medical providers fear that discussing this vaccine in the context of adolescent sexual activity may either provide the adolescent with an impression that sexual activity at their age is condoned, or may raise conversational topics that parents and/or providers may not be comfortable addressing. In response to this concern, many have advocated focusing HPV vaccine discussions on cancer prevention, and minimizing the sexual aspects of the infection.

The recent IJHPR article by Velan and Yadgar et al [2] takes this concept a few steps further by describing the desexualization of HPV vaccination. They define this process as a) purposefully hiding information that HPV is sexually transmitted, b) blurring information about the sexual nature of HPV (i.e. it is transmitted “skin to skin”), or c) distancing the time of HPV vaccination as far away from the time of likely sexual debut as possible. They argue, using ethical principles as a framework, that this approach to HPV vaccination may be harmful. They acknowledge that for some populations a desexualization approach may actually *increase* levels of vaccine uptake, while contending that on balance there are more people who would be harmed by such a strategy. Examples include adolescents whose parents tend to be passive about vaccination decisions, whose parents feel discussing sex with their adolescent is important, and adolescents that are homosexual.

As a practicing pediatrician, and one that has studied HPV vaccine utilization extensively, I have had many conversations with parents and their families about this vaccine – some successful and some not. Given this background, I found the article quite interesting. The authors raise several powerful points about why desexualization could be very harmful. For example, when the public perceives that authorities are withholding information (in this case that HPV is primarily transmitted sexually), this can significantly undermine trust, and therefore enthusiasm, about the vaccine. In a time of growing vaccine hesitancy for all vaccines [3], this concern is especially salient. Also compelling is the notion that failing to acknowledge the sexual nature of HPV breeches adolescent autonomy – shouldn’t they know what it is they are receiving and why?

Yet, as the authors rightly point out, the balance of harms and benefits to desexualizing HPV vaccination is not clear cut, and in some cases desexualizing the vaccine can be advantageous. For example, among parents whose discomfort about discussing sex in the context of their adolescent is strong, or who strongly feel their adolescent will not be at risk for HPV exposure at any point in their lives (regardless of whether this perception is true or not), de-emphasizing the sexual aspect of HPV infection and focusing instead on cancer prevention can be quite useful. In my practice, I personally have encountered such situations many, many times.

So where does that leave us? In my mind, the best approach falls squarely in the middle, and reflects what I have found most successful in my own clinical experience. First, we cannot actively hide the fact that HPV is a sexually transmitted infection. I believe that it is critical to emphasize that becoming infected with HPV is the norm, not the exception. With nearly 50% of individuals infected (in the US) with HPV at any given time [4] (Fig. 1), and most being infected at some point in their lifetime [5], is the rule rather than the exception. This is true if you are straight or gay, have one partner or many, or live in highly conservative or highly liberal populations. In fact, I have heard colleagues suggest we refer to HPV as “normal flora,” much like we do for staphylococcus or streptococcus as it relates to the skin, or *Escherichia coli* in the intestine. I love this approach – it sends the message that HPV is just a part of normal human experience, and therefore has no bearing on ones’ “behavior.” It tells the public what they need to know about how the infection is transmitted, yet does not overlay any judgement about each individual’s own personal risk for infection. Moreover, it paves the way forward for normalization of other vaccines that are likely to be available at some point in the future against other sexually transmitted infections such as HIV, Chlamydia and herpes. The more work we can do now to make vaccination against infections that are transmitted sexually a socially acceptable and routine practice, the better prepared we will be to accept these vaccines as a society if and when they become available in the future.

**Fig. 1:**
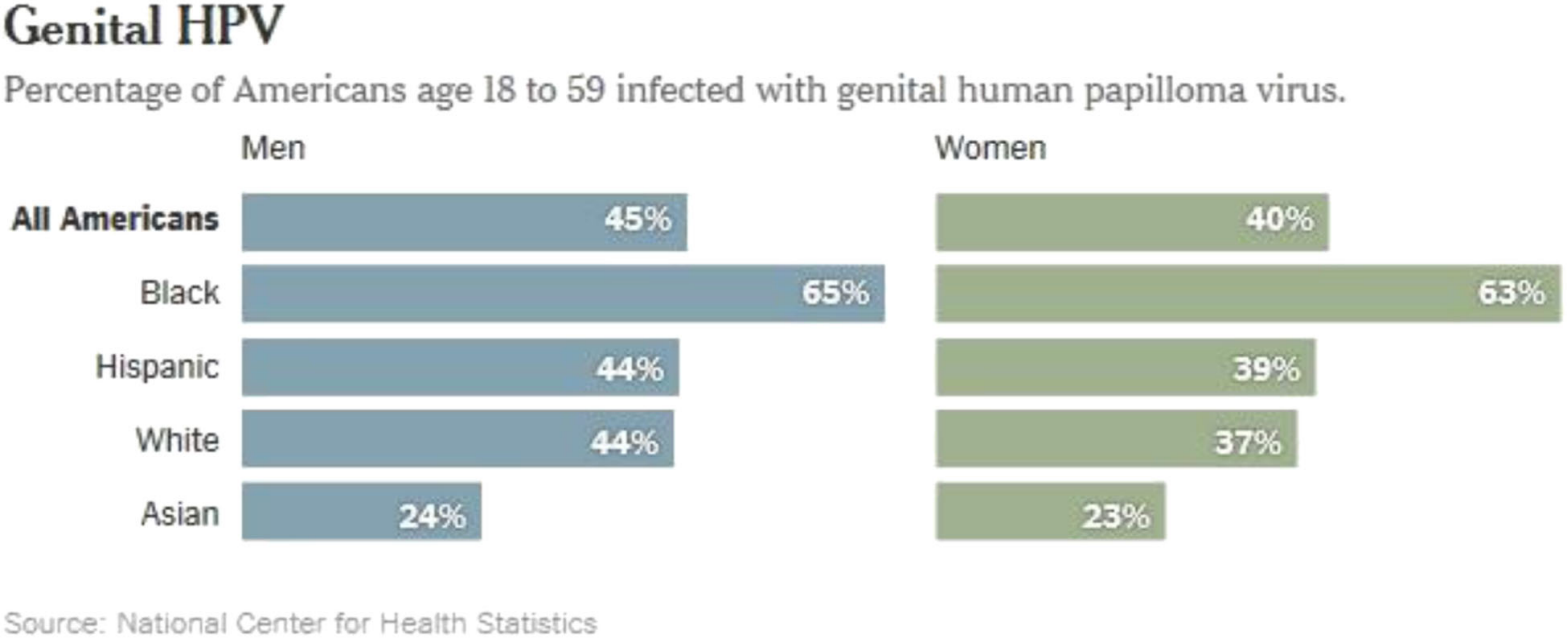
Prevalence of Genital HPV Infection Among US Men and Women, 2011–2014 [4]

So, while I believe it is critical to make clear the nature of HPV transmission, given how ubiquitous HPV infection is, I am a firm believer in also de-emphasizing the sexual aspects of HPV. Not only does this approach represent my own small attempt to normalize HPV infection for my patients, but I also disagree with many of the concerns raised by the authors about why such an approach may be harmful. There was a lack of references to scientific studies to provide evidence for several of their arguments, suggesting their conclusions were largely a matter of opinion. As such, I find it difficult to place these arguments over that of my own experience and that of my pediatric colleagues.

One such argument that was posited by the authors is that when HPV vaccination is desexualized, it “aborts” liberal parents’ opportunities to engage in a discussion about sex with their adolescent. This has definitely not been my own experience in practice. Instead, the opposite is often true – these are the parents that have typically *already* had these discussions with their children by the time they are eligible for HPV vaccination! In these parents’ minds HPV vaccination is an extremely minor, or even irrelevant, part of their overall approach to educating their children about sexual health.

Another rationale given in the article for the potential harm in desexualizing HPV vaccine discussions is that it obviates the opportunity to discuss the “dangers of male homosexuality” with adolescent boys (and their parents) that may have these sexual inclinations. Yet, in my mind the two are not linked. First, as the authors point out, most adolescents are not well solidified in their sexual orientation during early adolescence, making it a potentially harmful situation to “label” someone as such when they may still be trying to figure themselves out. Moreover, the entire issue of gender identification and sexual orientation is becoming increasingly complex as more and more individuals express a variety of gender identity and sexual orientation permutations [3]. Instead, I believe that information about the potential danger from *all* types of sexual contact need to be systematically addressed and provided to all adolescents, and that these conversations should occur irrespective of any conversations about HPV vaccination. It is our duty as parents, teachers and medical practitioners to make sure our children are knowledgeable about *all* sexually transmitted diseases. Even in highly conservative societies, this information is critical for ensuring that, even if not personally relevant, correct information can be conveyed by adolescents to their peers, and eventually to their own children.

## Conclusions

To put it succinctly, I agree it would be highly harmful to purposefully try to obscure the fact that HPV is sexually transmitted. Doing so could have serious negative consequences for the public’s increasing skepticism about vaccination. Yet I believe that parent/provider discussions about vaccination *should* de-emphasize sex and focus more on cancer prevention. Discussions about how to protect oneself against HPV *exposure* need to be provided in the broader context of overall sexual health and sexually transmitted disease prevention. Emphasizing the sexual transmissibility of HPV infection during the vaccination discussion gives the wrong message to parents by perpetuating the HPV vaccination is only useful for “at risk” individuals, and undermines the reality that essentially everyone is at risk for HPV at some point in their lives. It is my opinion that thinking of HPV as “normal flora” that happens to be transmitted mostly sexually, instead of a “sexually transmitted infection” is critical to shift the thinking of parents, adolescents and society as a whole. Having providers normalize exposure to this infection as an essentially unavoidable party of everyday life could go a long way in helping us as a society realize the full cancer preventing benefits of this remarkable vaccine.

## Abbreviations

HIV: human immunodeficiency virus
HPV: Human papillomavirus
US: United States
